# Sustainable development and health assessment model of higher education in India: A mathematical modeling approach

**DOI:** 10.1371/journal.pone.0261776

**Published:** 2021-12-28

**Authors:** Chenchen Deng, Song Yang, Qingyang Liu, Songjie Feng, Chuangbin Chen

**Affiliations:** 1 Jinhe Center for Economic Research, Xi’an Jiaotong University, Xi’an City, Shaanxi, China; 2 School of Energy and Power Engineering, Xi’an Jiaotong University, Xi’an City, Shaanxi, China; 3 School of Electrical Engineering, Xi’an Jiaotong University, Xi’an City, Shaanxi, China; 4 Graduate School of Engineering, Tokyo University of Agriculture and Technology, Koganei City, Tokyo, Japan; University of Western Australia, AUSTRALIA

## Abstract

The Coronavirus Disease 2019 has resulted in a transition from physical education to online learning, leading to a collapse of the established educational order and a wisdom test for the education governance system. As a country seriously affected by the pandemic, the health of the Indian higher education system urgently requires assessment to achieve sustainable development and maximize educational externalities. This research systematically proposes a health assessment model from four perspectives, including educational volume, efficiency, equality, and sustainability, by employing the Technique for Order Preference by Similarity to an Ideal Solution Model, Principal Component Analysis, DEA-Tobit Model, and Augmented Solow Model. Empirical results demonstrate that India has high efficiency and an absolute health score in the higher education system through multiple comparisons between India and the other selected countries while having certain deficiencies in equality and sustainability. Additionally, single-target and multiple-target path are simultaneously proposed to enhance the Indian current education system. The multiple-target approach of the India-China-Japan-Europe-USA process is more feasible to achieve sustainable development, which would improve the overall health score from .351 to .716. This finding also reveals that the changes are relatively complex and would take 91.5 years considering the relationship between economic growth rates and crucial indicators. Four targeted policies are suggested for each catching-up period, including expanding and increasing the social funding sources, striving for government expenditure support to improve infrastructures, imposing gender equality in education, and accelerating the construction of high-quality teachers.

## 1. Introduction

The Coronavirus Disease 2019 (COVID-19) has profoundly impacted humanitarian and economic fallout by reducing employment opportunities and high levels of restrictions on education resources [1]. Consequently, many commercial and educational activities have stagnated, and people face tremendous challenges. From June 15, 2021, a total of 175,987,176 confirmed cases of COVID-19 had been reported in 223 countries/regions, causing 3,811,561 fatalities [2]. India, the world’s second-most populous country, is mainly affected by the COVID-19 outbreak, and its new daily infections began increasing in April 2021. According to the Indian Ministry of Health and Family Welfare (MHFW), 28,388,100 confirmed cases of COVID-19 and 379,573 Indian deaths were confirmed on June 15, 2021 [3]. COVID-19 has provoked an unprecedented revolution in the overall education system, forcing the closure of many universities and the collapse of investment in endowment funds [4]. In India, rural students and teachers face significant bottlenecks on internet access, electricity supply, and technical processing skills [5]. The gap between the rich and the poor and the gap between urban and rural areas are becoming increasingly critical.

To alleviate the impact of the epidemic on the Indian higher education system (India-HES), the government has implemented an expansionary fiscal policy and structural adjustment of educational resources to address the short-term and long-term challenges faced by education. However, these strategies have had little effect on alleviating the fortunes of India-HES because there are four types of universities in India (Central, State, Deemed, and Private). Their structure is complex, and the increase in affiliated colleges makes academic governance increasingly challenging to manage [6]. Simultaneously, most India-HES are located in metropolitan regions, and most Indian students attend 574 universities 35,500 affiliated colleges, the majority of which receive low-quality education [7]. Empirical studies demonstrate that approximately one-quarter of college students in India have low and middle-level knowledge of preventing neo-coronary pneumonia, negative attitudes, and poor behavior [8]. Within the current structure of India-HES, restricted by students’ cognitive level, there is much resistance to implementing epidemic prevention and control.

Although India has a high volume of HES, it also faces challenges in admissions, equity, quality, infrastructure, teachers, privatization, research, and innovation [9]. The long-standing social contradiction is particularly evident in HES, and the efficiency and sustainability of India-HES have also been controversial issues for a long time. Inequality in Indian society has traditionally revolved around castes and the corresponding untouchable relationships and economic wealth, land rights, and employment status [10]. In this process, education is a medium for transmitting inequality and realizing cultural reproduction. Low-income families could hardly achieve class crossover through education [11]. Moreover, evidence from HES diversion options in India suggests varying degrees of gender segregation in the educational diversion at Indian universities, which is not due to individual gender differences, but institutional and systematic factors [12]. Gender inequality in education is most common in northern India [13].

In recent years, despite various measures introduced by the Indian government to promote gender equality in HES, the multifaceted educational administration system and the patrilineal family system continue to constrain the resolution of gender inequality. According to the United Nations Gender Inequality Index, India ranks 131 out of 189 countries [14]. Meanwhile, the unique caste system of Indian society is also evident in the field of education. While affirmative action plans for low caste groups in Indian engineering schools have become a favorable policy for increasing class diversity and allocating resources to relatively vulnerable families, individuals with solid family economic and cultural capital benefit most from retention policies [15]. Based on this situation, the Indian government has enacted the National Education Policy to alleviate these challenges, aiming to prepare students to become active contributors to the Fourth Industrial Revolution [16]. However, the effectiveness of policy implementation depends on a wide range of stakeholders, and its sustainability in promoting India-HES has not yet been empirically tested.

**Fig 1:**
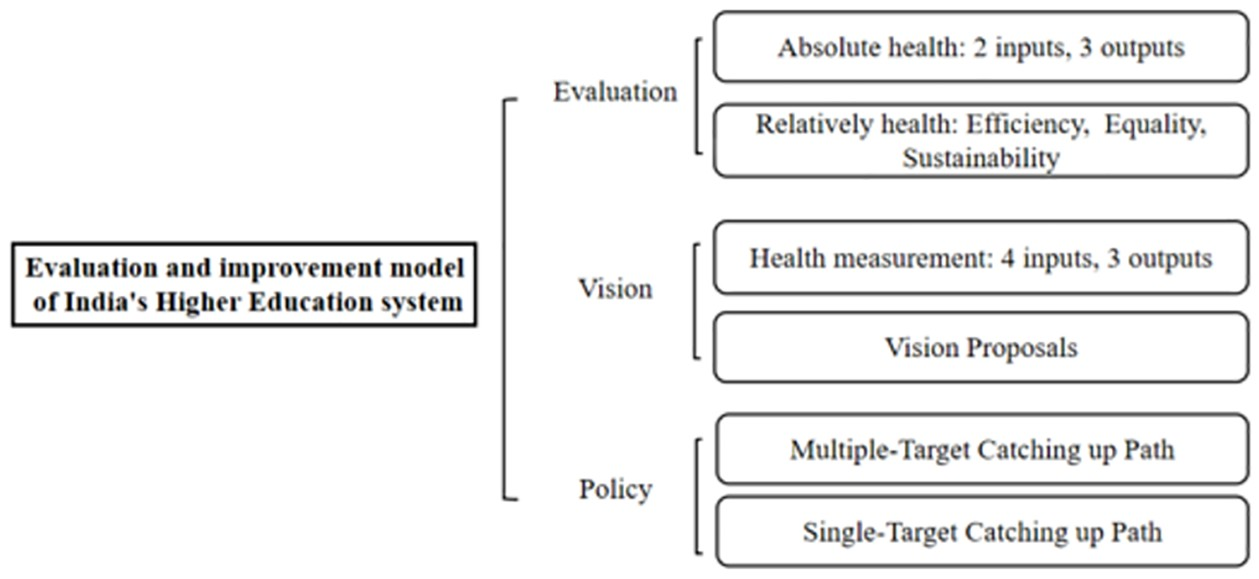
Overview of the present study.

This research aims to identify several crucial indicators for quantifying the system’s health and construct models for health assessment based on the background. Through multiple comparisons between India and other countries, a fundamental overview of India-HES is evaluated, and an overview of this research is displayed in Fig 1. Following this, we provide an achievable and reasonable vision for the system and construct models to measure the health of both the current and proposed tertiary education system. Furthermore, targeted policies and implementation schedules are put forward to support the transition from the present to the proposed state. Finally, analyzing the potential impacts of the proposed suggestions in the transition to completion from different perspectives is our primary concern, demonstrating that changes are somewhat tricky.

## 2. Literature review

India is the world’s second-most populous country, with limited educational resources and a great demand for education. This contradiction is fundamental, leaving a sequence of issues to be addressed [17]. The quality and quantity of India-HES have changed radically since the 1980s, particularly spatial distribution, diversity, and multi-dimensional changes [18]. India-HES consists of four levels: a few select universities at the top, most universities at the second level, most of the academy of sciences at the third, and vocational education colleges at the bottom [19]. Meanwhile, the public policy in India-HES has been assessed, and relevant insights into Indian politics, policies, and philosophy of India-HES have been provided [19]. Large-scale Indian engineering students have immigrated from India to the USA seeking post-graduate education, which inadvertently undermined India-HES [20]. Rajkhowa (2013) investigated the challenges and opportunities of cross-border HES, helping to address problems associated with the growth of cross-border India- HES and putting forward new challenges and opportunities of cross-border HES [21]. García-Arroyo (2017) establishes a market-oriented India-HES directly affecting Indian women and jeopardizes their academic quality and gender equality [22]. Altbach (2014) discussed the international recognition of the Indian Institute of Technology and related institutions and considered the challenges facing India-HES in the 21st century [7].

Based on the current challenges of India-HES, several researchers have proposed improvements from distinct perspectives. Sujlana (2017) suggested that the Indian government improve the existing Indian education policy to reduce bias toward underprivileged students [23]. Chand and Arora (2008) proposed creating the Infonet Digital Library Alliance organized by the Indian University Funding Committee [24]. Mukesh et al. (2018) analyzed the entrepreneurial potential of university students and the teaching of entrepreneurship in India-HES [25]. Baily (2015) explored how India-HES policies systematically reach certain marginalized students [26]. The objective was to explore how the managers of HES view the growth of this partnership to change the status quo of privilege and power. Gupta and Sengupta (2021) demonstrated that the development of science and technology would bring about a quality improvement of India- HES [27].

The COVID-19 had profound impact on India-HES. Sood and Sharma (2020) [28] investigated factors affecting students’ psychological well-being in HES during the COVID-19. Baloran (2020) indicated that the COVID-19 could aggravate anxiety among students. Through examining students’ knowledge acquisition and attitudes, Baloran (2020) also suggests that addressing the mental health of college students during the pandemic is in urgent need [29]. Padmanaban et al. (2021) suggested ways to mitigate the effects of a pandemic spreading among students to help India-HES transition smoothly [8]. Chakraborty et al. (2020) demonstrated that dental students and practitioners had more severe depression and made relevant suggestions for their psychological health [30]. Based on the interaction between stress and the response theory, Sood and Sharma (2020) used the least-squares structural equation model to explore factors influencing students’ mental health [28]. Kumar et al. (2020) proposed a program examining the experience of college teachers using webinars as a teaching tool for engineering students [31]. Saha et al. (2021) discussed the challenges faced by HES learners and institutions in India. In particular, online learning was studied and changing future education paths during pandemics [32]. Geng et al. (2020) presented a study of *7E* indicators and proposed sustainable HES development strategies suitable for different regions [33]. According to Sultan and Tarafder (2007), the absolute health aspect of HES consists mainly of educational volume. The relative health aspect includes the efficiency, fairness, and sustainability of education [34].

Meanwhile, after the large-scale outbreak of the epidemic in 2021, the sustainable development of India-HES is also challenged, but this part of the research is relatively insufficient. Overall, previous literatures have examined the primary potential factors that affect the health of India-HES. However, these studies constitute a unilateral evaluation of the Indian sustainable health education system and lack a comprehensive and systematic research methodology. Furthermore, the relevant existing studies only evaluate a single advanced country but lack concern towards less developed states to realize sustainable development in India-HES.

To narrow the remaining gaps, this research systematically proposes a health assessment model from four perspectives, including educational volume, efficiency, equality, and sustainability, by employing the Technique for Order Preference by Similarity to an Ideal Solution Model (TOPSIS), Principal Component Analysis, DEA-Tobit Model, and Augmented Solow Model. Simultaneously, we compare the educational systems of India with the United States, Europe, Japan, and China, proposing policy recommendations for India-HES.

## 3. Data and methodology

### 3.1. Data and assumptions

The data is collected from the Organization for Economic Co-operation and Development (OECD) database and the World Bank database. According to the University ranking and tertiary education system benchmarking, eight countries are selected with different levels of educational advancement. The first tier includes the United States; the second tier is Norway, Belgium, and Australia; the third tier comprises Japan; the fourth tier is Russia and China; the fifth tier is India.

To simplify the model, two crucial assumptions are made to facilitate the following discussion. First, this research assumes the government only implements policy changes for a single evaluation indicator of HES at one period, meaning that if there are multiple indicators to be improved in a certain period, the government will enhance each indicator in turn. The duration of this period is the sum of the time of enhancing each indicator. Second, the economic capacity of a state is sufficient to support the effective implementation of policies aimed at education development, irrespective of war and emergency wartime situations resulting from severe epidemics. Therefore, the variable descriptions are presented as follows in Table 1, and the original sources of the data are shown in S1 Table.

**Table 1:** Abbreviation meaning and symbolic description.

Abbreviation	Meaning
DEA	Data envelopment analysis
CCR	A traditional type of DEA model
DMU	Dynamic model update
TOPSIS	Technique of order preference by similarity to an idea solution
PCA	Principal Component Analysis
QS300	QS World University Rankings top 300
R&D	Research & Development
GDP	Gross Domestic Product
7E	A model examination the health of the whole education system
OECD	Organization for Economic Co-operation and Development
*α*	Capital share of income
*β*	Human capital share of income
*h*(*t*)	Human capital per labor
*g*_*h*_	Growth rate of human capital per labor
*IN*	Input
*O*	Output
*X*	Value of input
*m*	Growth rate of input
*IN*_1_	Funding investment
*IN*_2_	Infrastructure/personnel investment
*O*_1_	Technology/economic entity output
*O*_2_	Social benefits
*O*_3_	Admission and employment rate

### 3.2. Framework of the assessment system

To evaluate the health of the entire India-HES, this paper determines indicators that quantify and reflect the reality. The *7E* framework as an absolute path are used to analyze this issue: equality, expenditure, equity, economy, effectiveness, efficiency, and existence [33]. However, dimensions contained in our framework are still too general and lack pertinence for evaluating HES health. In this research, the health connotation of the tertiary education system is divided into absolute and relative health. To construct the assessment system, this article takes the volume of India-HES as an evaluation of absolute health. It takes the efficiency, equality, and sustainability of HES into account to measure relative health.

#### 3.2.1. Absolute health evaluation

As a relatively sophisticated system with various components contained, the absolute health of a country’s HES is analyzed firstly. Additionally, absolute health is defined by the volume of a specific HES. Next, the data is divided into input and output indicators, with two and three dimensions included. Furthermore, TOPSIS is used to achieve data dimensionality reduction. Finally, to rank the absolute volume of HES, this paper adopts the Principal Component Analysis (PCA) to conduct further discussions. The basic idea behind PCA is shown, and we suppose *Z*_*i*_ as the *i th* component.

Z1=c11X1+c12X2+⋯+c1pXpZ2=c21X1+c22X2+⋯+c2pXp⋮Zp=cp1X1+cp2X2+⋯+cppXp (1)

where $c_{i1}^{2}+c_{i2}^{2}+\cdots +c_{ip}^{2}=1$, and [*c*_*i*1_, *c*_*i*2_, ⋯, *c*_*ip*_] maximizes Var(*Z*_*i*_).

#### 3.2.2. Relative health evaluation

*3*.*2*.*2*.*1*. *Efficiency*. Efficiency is defined as a measurement of whether sustainable HES interacts and is effectively implemented. The efficiency evaluation of HES is further complicated because these institutions have multiple inputs and outputs, and not all these outputs can be measured from an economic perspective. Therefore, we adopt the DEA model, using the Pareto optimal boundary in the economic sense to measure input and output efficiency in HES. Additionally, the econometric Tobit regression model is adopted to analyze the impacts of several external factors on the efficiency performance of HES.

The CCR model (A traditional type of DEA model) could solve inefficient decision-making units. However, the efficiency values of the decision-making units on the frontier are all 1, meaning multiple units are relatively effective simultaneously. Due to the limitation of the traditional CCR model that cannot rank the efficiency of DEA-effective Dynamic model update (DMU), this research employs the Super-Efficiency model to solve it.

The Super-Efficiency model removes the constraint of *j*0 in the CCR model, which could obtain an efficiency value greater than or equal to 1 for the effective decision-making unit *j*0 (if valid). The basic model is as follows:

$$min\;\left[\theta -\varepsilon \left(\sum_{j=1}^{m}s^{-}+\sum_{j=1}^{r}s^{+}\right)\right]$$ (2)

$$\text{s.}\;\text{t.}\;\left\{\begin{array}{c} \sum_{\begin{array}{c} j=1\\ j\neq j_{0} \end{array}}^{n}x_{j}\lambda_{j}+s^{-}=\theta x_{0}\\ \sum_{j=10}^{n}y_{j}\lambda_{j}-s^{+}=y_{0}\\ \lambda_{j}\geq 0,j=1,2,\cdots ,n\\ s^{+}\geq 0,s^{-}\geq 0 \end{array}\right.$$ (3)

Where *θ* is the efficiency evaluation index of the decision-making unit (*DMU*_*j*0_). *x*_*j*_ is the set of input elements for DMU_*j*_. *y*_*j*_ is the set of output elements of DMU_*j*_. *λ*_*j*_ represents the combination proportion of DMU*j*, *s−*, *s*+ are slack variables. *x*_0_ and *y*_0_ are input vector and output vectors separately. The economic implications are:

1. When *θ* = 1 and *s*^*−*^ = *s*^+^ = 0, *DMU*_*j*0_ is called DEA efficient; namely, in the economic system composed of n decision-making units, when the output *y*_0_ obtained based on the original input *x*_0_ has reached the optimal value.
2. When *s*^*−*^ ≠ 0 or *s*^+^ ≠ 0, then *DMU*_*j*0_ is said to be weak DEA efficient; namely, in the economic system composed of *n* decision units, the input *x*_0_ is reduced, and the original output of *y*_0_ can be kept unchanged, or the output is increased when input *x*_0_ is constant.
3. When *θ*< 1, *DMU*_*j*0_ is non-DEA efficient.

*3*.*2*.*2*.*2*. *Equality and sustainability*. Equality reflects coordinated participation of the public in HES activities and the coordinated contribution of HES to society, including aspects of gender, race, and social status. To measure the equality dimension of HES, this paper applies TOPSIS to achieve data dimension reduction. Besides, the sustainability dimension of a system indicates maintaining its effectiveness over time. For the sustainability dimension, we employ an Augmented Solow Model to calculate the growth rate of human capital in the equilibrium state [35] to measure the sustainability of the tertiary education system.

## 4. Result

### 4.1. Absolute health evaluation

**Fig 2:**
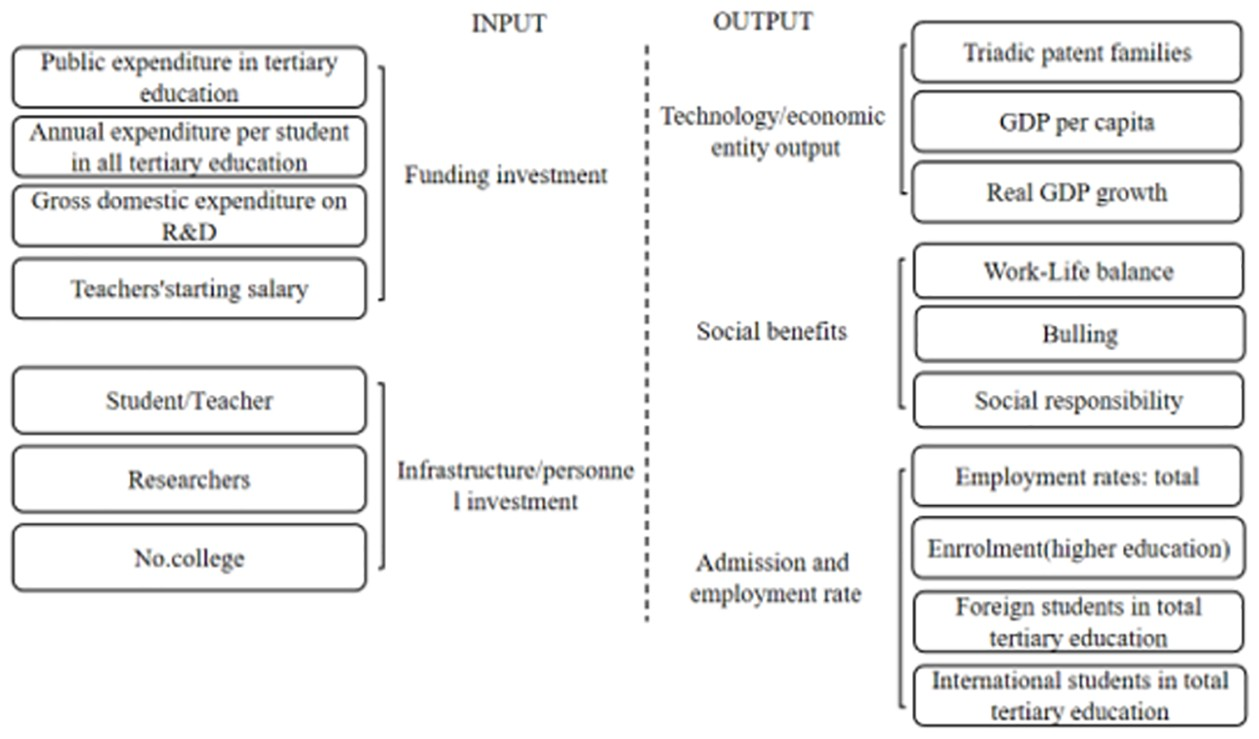
Input and output indicators.

**Fig 3:**
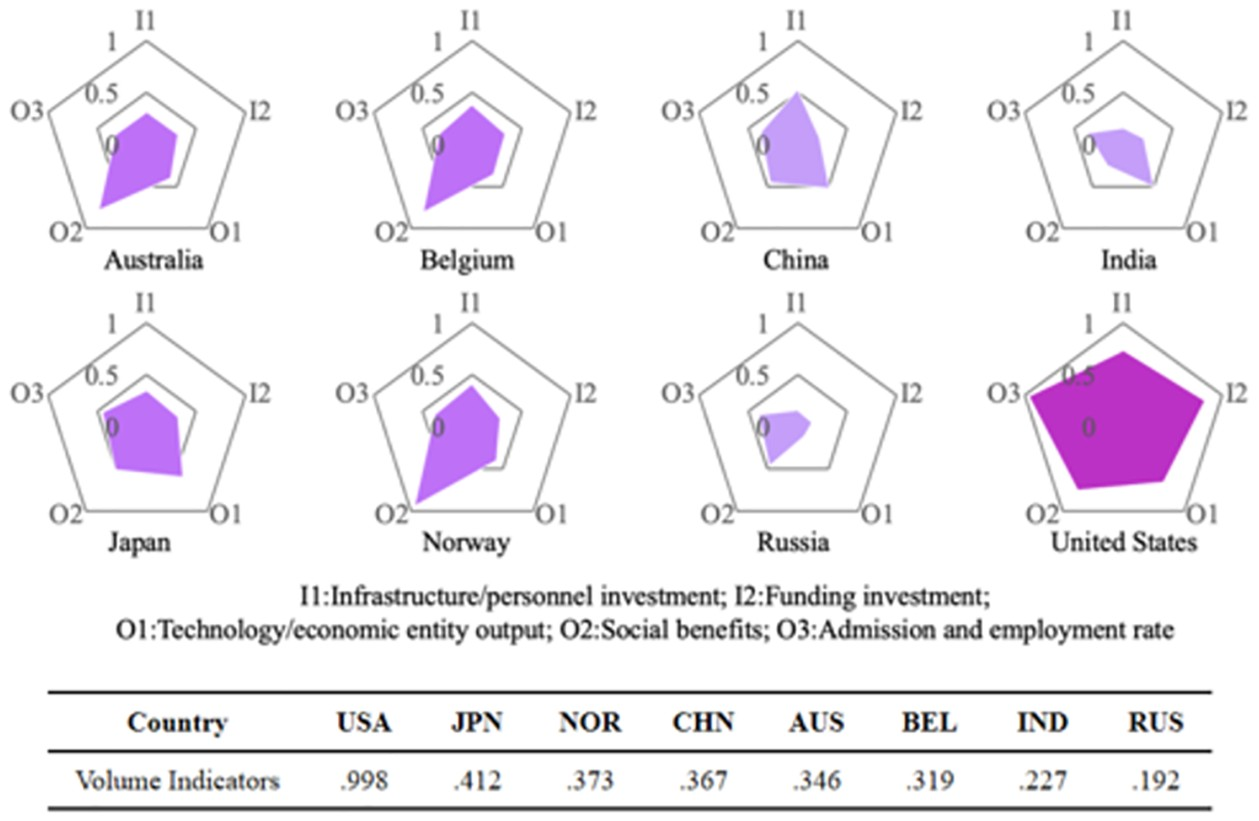
Absolute health ranking result.

HES is a complex activity, including a variety of inputs and outputs in the education system. To implement the following analysis, we divide data into input and output indicators and apply TOPSIS to achieve data dimension reduction and get five indicators, including two input indicators C_1_, C_2_, and three output indicators O_1_, O_2_, and O_3_, separately in Fig 2. To rank the absolute volume of the HES, this research adopts the PCA to conduct decisions, and the result is shown in Fig 3. The countries with the highest to lowest scores of absolute HES volume are the United States, Japan, Norway, China, Australia, Belgium, India, and Russia, whose scores are .999, .411, .373, .368, .346, .319, .227, and .192, respectively. Thus, the above volume indicators are consistent with our consensus.

### 4.2. Relative health evaluation

#### 4.2.1. Efficiency dimension

Table 2 presents values of *θ* based on selected indicators, which demonstrate the efficiency of different HES. Countries with the top three input-output ratios are Russia, China, and India, meaning their HES are rising. The fourth to seventh states are Norway, Australia, Japan, and Belgium, while the United States ranked last. Although the input and output indicators of the countries with relatively high ranks are not outstanding, they still get a higher score due to their highly efficient ratio of converting input into output. Notably, the United States has an absolute advantage in all input and output indicators; namely, all its indicators are in the leading position in the world. However, this does not necessarily mean a high efficiency in HES, which may be because when the input and output of education and science and technology have reached a specific target, more investment is needed to improve efficiency accordingly.

**Table 2:** DEA efficiency.

Country	IND	RUS	CHN	NOR	AUS	JPN	BEL	USA
DEA efficiency	1.875	1.638	1.169	1.110	.978	.844	.843	.544

On this basis, Table 3 performs Tobit regression results. In the first phase, this research replaces all the input variables and output variations with the first phase of the DEA-Tobit model to evaluate the efficiency of converting input variables into output variables in different countries. After the efficiency score is obtained, the Tobit model is employed to perform the resulting efficiency indicators for Tobit regression of the environmental variables. For the second stage of Tobit regression, all three external variables are almost statistically significant under the 10% significance level. The coefficient of GDP per capita has a significant negative value, meaning that relatively more prosperous states would achieve lower efficiency of education because the more endowment a state has, the less likely it will reach equivalent efficiency. This corresponds to the basic concept of economic growth theory that states lower initial values of the capital per labor have higher per capita growth rates and will catch up or converge to those with higher capital-labor ratios and finally reach zero growth rate [36]. Furthermore, the coefficient of population-level is negative, corresponding to common sense that the limited education resources would contradict infinite education demand, leading to a less efficient education system. Besides, this research also observes a positive coefficient of population growth, which is reasonable for more dynamics and energy that younger generations contribute to the economy and education system.

**Table 3:** Tobit regression result.

θ	coefficient	t	P>|t|
GDP per capita	-.001	-4.06	.010
popul-ls2013	-5.13e-7	-2.06	.094
popul-es2013	.271	2.00	.102
cons	2.207	6.47	.001

#### 4.2.2. Equality dimension

As a core principle in HES, equality includes various dimensions and is relatively hard to measure. However, the connotation behind education equality usually contains educational opportunity equality and equal rights to receive an education. Therefore, three indicators are considered simultaneously to construct an equality system of HES.

- SES: Standard error of the number of 15-year-olds per school computer, measuring the area equality.
- ERD: Enrollment Rate Difference for tertiary education by gender, measuring gender equality.
- PR: Poverty Rate, measuring social status equality.

Since the relationship between SES and ERD is negatively correlated with the measurement of PR, these two indicators are transformed by using the following equations:

$$SEST=\frac{1}{SES}$$ (4)

ERDT=ERD-1 (5)

This research again adopts TOPSIS to conduct a comprehensive evaluation and obtain a ranking system of the equality of HES.

Based on Table 4, the top three countries in education equality are European and Australian states, which concerns the lower gap between the rich and the poor. The fourth to eighth states are Russia, the United States, China, Japan, and India. With a score of -.566 in the assessment system, India presents the world’s most unequal HES. Considering the national situation of India, it is caused by the extreme imbalance of the ratio of men and women in HES. Additionally, the assessment score of China is -.466, which the relatively large poverty rate could explain. Similar logic could be applied to demonstrate the inequality of the United States.

**Table 4:** Equality of higher education.

Country	NOR	BEL	AUS	RUS	USA	CHN	JPN	IND
Equality	-.035	-.081	-.105	-.168	-.169	-.305	-.466	-.566

#### 4.2.3. Sustainability dimension

The sustainability of HES is a rather complicated issue in the educational sense. As the goal of HES, the capacity and quality of students who receive HES is the critical issue are concerned about. This problem is based on the growth rate of human capital, reflecting the growth of knowledge, skills, and health state of students receiving tertiary education. The higher the growth rate of human capital accumulation, the more sustainable a country’s HES is. This research devises an Augmented Solow Model to calculate the growth rate of human capital in the equilibrium state. Our model is:

$$Y\left(t\right)=K{\left(t\right)}^{\alpha }H{\left(t\right)}^{\beta }{\left(A\left(t\right)L\left(t\right)\right)}^{1-\alpha -\beta }$$ (6)

Where *Y*_*t*_ is the total output, *H*_*t*_ is human capital stock, *K*_*t*_ is financial capital input, *A*_*t*_ is technological progress, L_t_ indicates the number of labor, *A*_*t*_*L*_*t*_ together means total effective labor, α is the coefficient of elasticity of financial capital, and *β* is the coefficient of elasticity of human capital. We assume that *L* and *A* grow at a given exogenous speed *n* and *g* separately.


$$L\left(t\right)=L\left(0\right)e^{nt}$$ (7)

The growth rate of the economy depends on:

$$\dot{h}\left(t\right)=s_{h}y\left(t\right)-\left(n+g+\delta \right)h\left(t\right)$$ (8)

*y = Y/AL*, *k = K/AL*, *h = H/AL*, representing output, capital stock, and human capital per effective labor.

The long-term growth rate of human capital per capita is as follows:

$$g_{h}=\frac{\dot{h}}{h}=\frac{\dot{H}}{H}-n$$ (9)

The expenditure on HES and the expenditure on research are adopted as two instrumental variables for total human capital investment. The growth rates of these two indicators are *r* and *f*.

The fundamental equation of the Solow model (Eq 10) proves that the derivative of $\dot{h}/h$ concerning *h* is negative:

$$\partial \left(\dot{h}/h\right)/\partial h=s\cdot \left[f^{\prime }\left(h\right)-f\left(h\right)/h\right]/h<0$$ (10)

Furthermore, empirical research has proved an absolute convergence of capital accumulation in OECD homogeneous states, meaning states with lower initial values of capital per labor have higher per capita growth rates. It will catch up or converge with higher capital-labor ratios and finally reach zero growth rate [36]. The accumulation growth rates of human capital per student of our selected eight states are illustrated in Table 5.

**Table 5:** Sustainable growth in education and technology.

Country	BEL	CHN	USA	IND	NOR	AUS	RUS	JPN
Sustainability (%)	23.89	13.43	2.92	.498	-2.99	-5.613	-6.674	-7.542

The country with the highest ranking for sustainable growth in education and technology is Belgium, followed by China, the United States, India, Norway, Australia, Russia, and Japan. Belgium is a developed country with constantly increasing investment in education and science and technology sustainability. China’s indicators of sustainable growth are growing for the sake of its increasing attention to the development of science and technology and education in recent years. Notably, the United States has a growth rate because it has broken through the limitation of decreasing return to scale effect of traditional capital, and the whole country has gradually transformed into a state with rich human capital. Under the premise of being sufficiently developed, the investment in education and scientific research in the United States can be regarded as human capital with increasing return to scale, meaning the United States is on the path to achieving sustainable and perpetual growth. Although Japan, Russia, and other countries have a large enough volume, they cannot achieve sustainable development due to the failure to complete the transformation from physical capital to human capital, resulting in a negative growth rate.

### 4.3. Overall health evaluation

This paper first normalizes four indicators reflecting the absolute and relative health of HES, considering that the dimensions and units of these indicators are entirely different. Then, the equation is as follows:

$$x_{i}^{\prime }=\frac{x_{i}-x_{min}}{x_{max}-x_{min}}$$ (11)

After the normalization of the four indicators, this article assume that absolute health (volume) and relative health (efficiency, equality, and sustainability) share the same proportion in the overall assessment system. We assign them equal weights of .5 and .5 and weigh them in three minor aspects: efficiency, equality, and sustainability in the relative health indicators. Efficiency and sustainability are more closely related to the development of relative health. This research gives greater weight to these indicators than equality, with .2, .1, and .2 separately. The complete evaluation system of tertiary education is presented as follows:

$${\text{Evaluation}\;\text{Score}}_{i}=0.5\;{\text{Volume}}_{i}+0.2\;{\text{Efficiency}}_{i}+0.1\;{\text{Equality}}_{i}+0.2\;{\text{Sustainability}}_{i}$$ (12)

**Fig 4:**
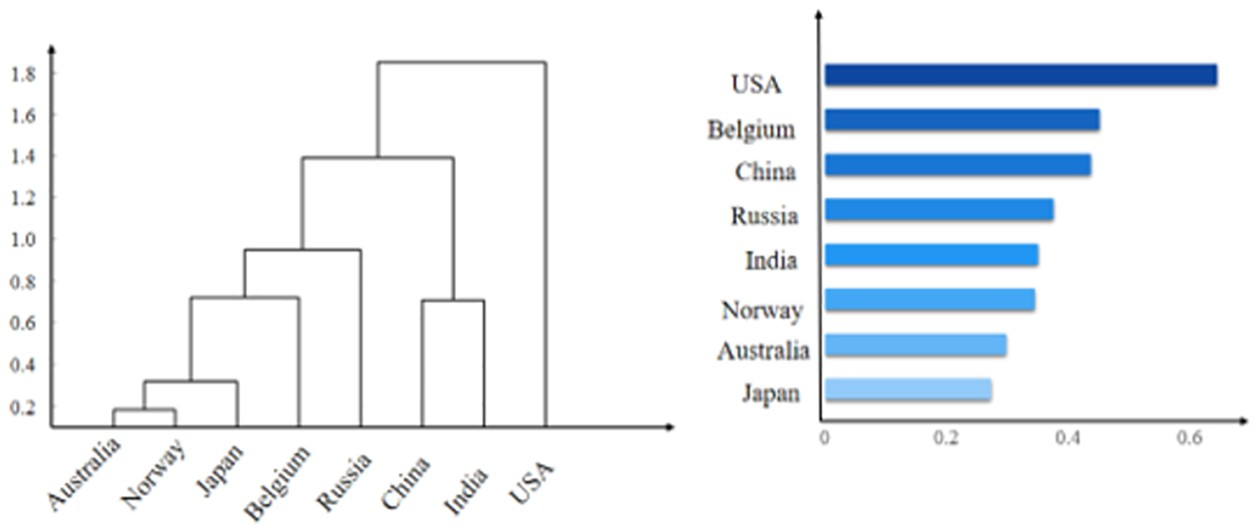
Result of cluster analysis.

The final ranking of the overall score for different states’ HES, as presented in Table 6. Additionally. This paper adopts Cluster Analysis, another multivariate statistical analysis method for the quantitative classification of multiple indicators to validate the proposed model. Fig 4 documents the analysis results.

**Table 6:** Overall score for different states’ higher education system.

Country	USA	BEL	CHN	RUS	IND	NOR	AUS	JPN
Overall score	.644	.451	.438	.377	.351	.345	.298	.273

## 5. Discussion

### 5.1. Health measurement model specification

The evaluation framework is concluded with the health score of HES as the final index. In this model, we still use evaluation scores to measure the health of our entire system. Combined with the variables we use to determine the efficiency and absolute volume of a country’s education system, we classify the above indicators according to the input and output of HES. IN_1_ represents funding investment, IN_2_ is infrastructure investment, IN_3_ displays the equality extent of country *i*, IN_4_ indicates sustainability, O_1_ is scientific or economic entity achievements, and O_2_ is social benefits and advanced education admission rate and employment rate, input determines output. The output indicators have solid practical significance.

Since the education equality level and education sustainability level of a state is the existing indicators of a country, we take them as the input to measure the national HES based on the original investment. In an economic sense, IN_1_ and IN_2_ are not significantly correlated. According to the linear relationship between scientific research and economic output and funding input in the economy, a multiple linear regression model is employed to infer the relationship between output and input, which is reasonable through our demonstration. Table 7 shows the results. The basic regression model is as follows:

$$y_{i}=\sum_{i=0}^{n}k_{i}\cdot x_{i}+e_{i}$$ (13)

*e*_*i*_ is the error term; *y*_*i*_ is the dependent variable; *x*_*i*_ is the independent variable. By using our regression equation, the following result is concluded:

$$\left[\begin{array}{ccc} a_{11} & \cdots & a_{15}\\ \vdots & & \vdots \\ a_{31} & \cdots & a_{35} \end{array}\right]\cdot \left[\begin{array}{c} IN_{1}\\ \vdots \\ IN_{i} \end{array}\right]=\left[\begin{array}{c} O_{1}\\ \vdots \\ O_{m} \end{array}\right]$$ (14)

**Table 7:** Multiple linear regression results.

	Expenditure	Infrastructure	Equality	Sustainability	Constant
Scientific/Economic	.632	.152	-.316	-.228	.356
Social	.098	.200	.532	-.045	.203
Educational	.169	.843	-.088	-0.88	.187

Because the final score for the efficiency and volume model is determined by IN_1_, IN_2_, OUT_1_, OUT_2_, OUT_3_, and OUT_1_, OUT_2_ and OUT_3_ are estimated by IN_1_ through IN_4_ through a multilinear regression; eventually, IN_1_ to IN_4_ are used to evaluate the efficiency and the volume model. Since the Evaluation Score is determined by Efficiency, Volume, IN_3_, and IN_4_ through weighted average, the final Evaluation Score can be determined by a form of function g from IN_1_ to IN_4_. The structure of the function is assumed in the following format:

$$\text{Evaluation}\;\text{Score}=g\left(IN_{1},IN_{2},IN_{3},IN_{4}\right)$$ (15)

According to our assumption, policy changes can directly affect our input variables, from IN_1_ to IN_4_, assuming that the values of the four input indicators of the current country are a_1_ to a_4_. The ideal input variables are b_1_ to b_4_. The value of the input index would be changed through a series of policy changes. After implementing a specific policy and after a particular time, we can change the initial input variable into the final ideal input variable.

Since TOPSIS and DEA-Tobit algorithms are used to measure volume and efficiency, the final evaluation score and input indicators have not been simply linear. Therefore, the final evaluation score by changing the value input variables. According to the final fitting process, the impact of the change of a unit input variable on the final score is concluded.


$$\text{Efficiency}=DEA\{\begin{array}{c} {IN}_{1}\;{IN}_{2}\;O_{1}\;O_{2}\;O_{3}\} \end{array}$$ (16)

$$\text{Volume}=PCA\{\begin{array}{c} {IN}_{1}\;{IN}_{2}\;O_{1}\;O_{2}\;O_{3}\} \end{array}$$ (17)

Score=0.5Volume+0.2Efficiency+0.1Equality+0.2Substainability (18)

India has been chosen as the state that has room for improvement. The empirical results suggest Belgium has the highest score regarding indicators of educational equality and educational sustainability. Regarding the volume score, the United States has an absolute advantage. Therefore, the ideal state is that India will eventually achieve Belgium’s educational equality and educational sustainability through a series of policy changes, with the United States’ volume characteristics. Specifically, our visions are to adjust the education input of India to the funding investment and the infrastructure investment of the United States and reach the state of education equality and sustainable education investment of Belgium. The current and proposed input values are shown in Table 8.

**Table 8:** Changes in the current value of each higher education indicator and our proposed value.

	IN_1_	IN_2_	IN_3_	IN_4_
Current value	.160	.206	0	.256
Proposed value	.740	.825	. 912	1

### 5.2. Policy recommendations

To settle several vital issues of concern and achieve our visions, two feasible paths are proposed to optimize the health indicators of India-HES.

#### 5.2.1. Single target catching up path

**Fig 5:**
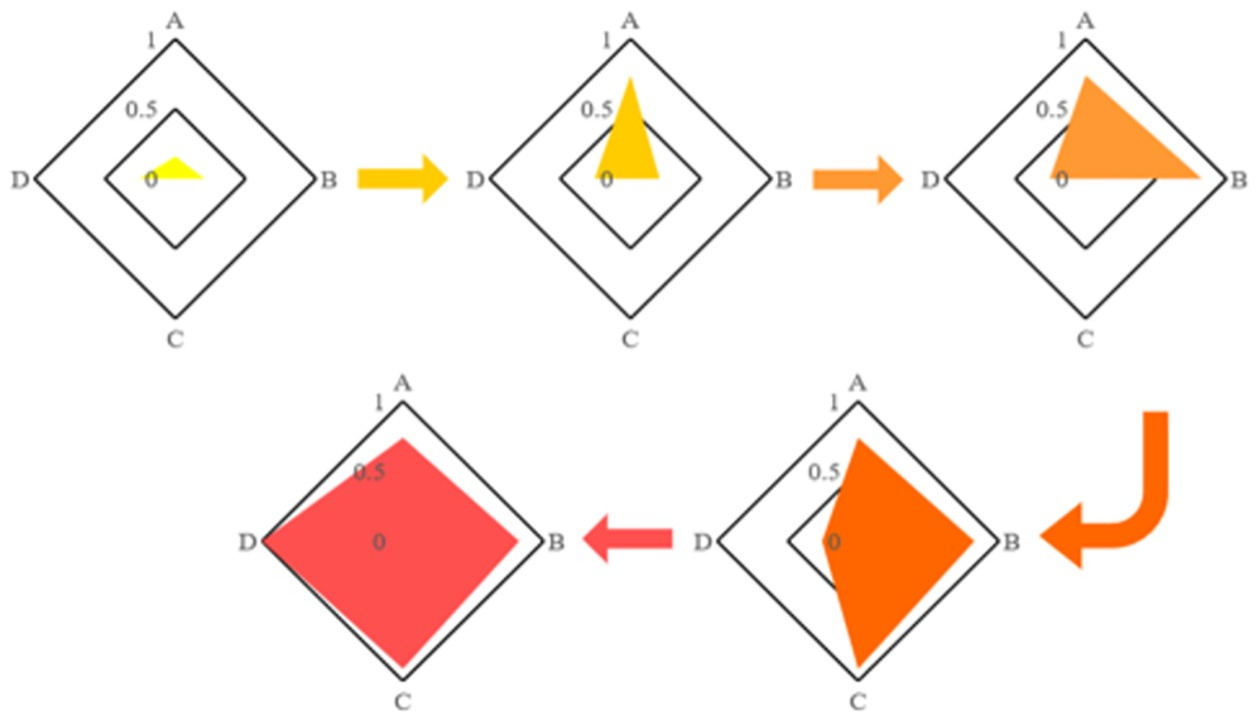
Transformation of the input value. A:Funding investment; B:Insfrastructure/personnel investment; C:Equality; D:Sustainability.

The first path is developing the Indian input indicators directly to the ideal health system input through policy changes shown in Fig 5. Since the perfect steady state indicators are fixed, the established order to change the indicators will not affect the final score and the final state’s time. Therefore, the change of the order is IN_1_, IN_2_, IN_3_, and IN_4_ are supposed. The transformation of the following indicators is the process followed by India in Table 9.

**Table 9:** The transformation of the output value.

Stage	Equality	Sustainability	O_1_	O_2_	O_3_
India	0	.256	.567	.403	.501
Stage1	0	.256	.796	.305	.462
Stage2	0	.256	.891	.429	.983
Stage3	1	.256	.602	.914	.904
Stage4	1	1	.433	.881	.838

Based on the results, the Indian health score change is followed by the continuous input change in Table 10. The monotonous increase in India’s total health score displays that the health system of Indian education is increasing, and it reaches the proposed ideal state.

**Table 10:** Transition of health value.

	India	Stage1	Stage2	Stage3	Stage4
Health	.351	.492	.499	.651	.716

#### 5.2.2. Multiple targets catching up path

To eliminate the drawback of the above path, the issue from a more micro perspective is analyzed. As a state pursuing the maximization of health indicators, this paper assumes that India optimizes indicators by steps and stages. Therefore, we divided the level of the tertiary education system into four echelons based on relevant literature. More specifically, the period for India to catch up with China, Japan, Belgium, and the United Stated separately are calculated.

**Fig 6:**
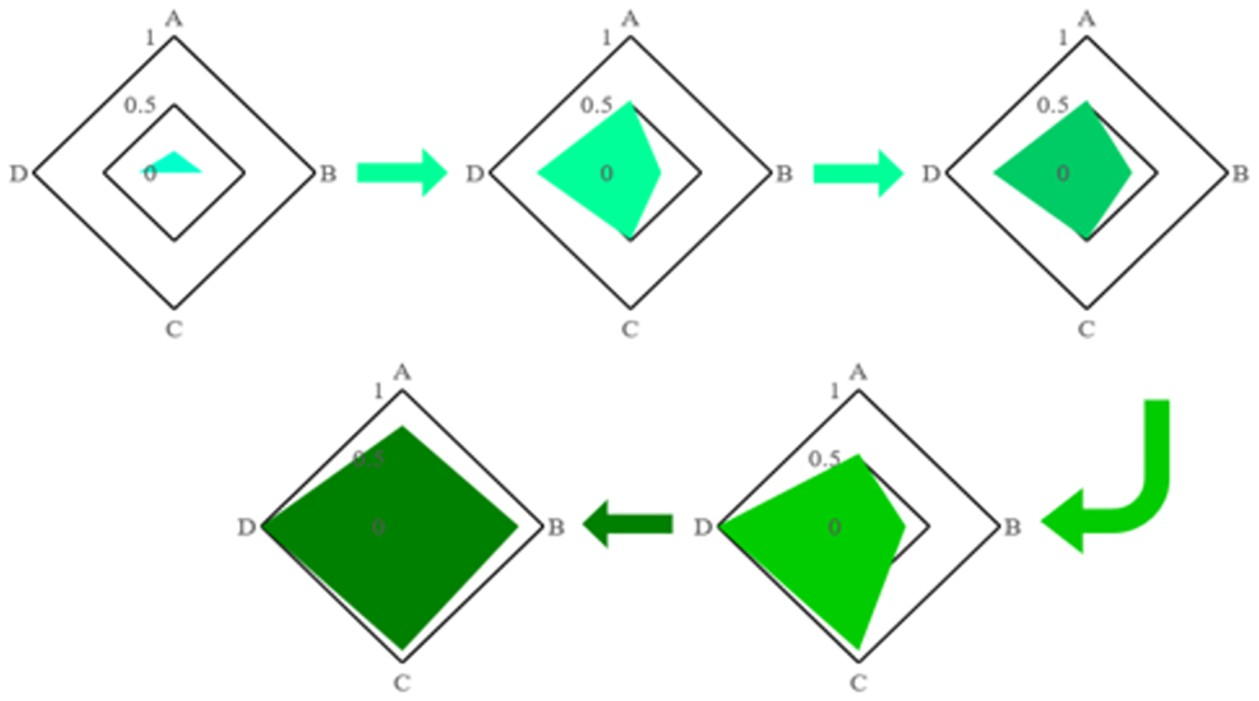
Transformation of the input value. A:Funding investment; B:Insfrastructure/personnel investment; C:Equality; D:Sustainability.

Our policy recommendations are linked to parameters that have a gap between India and the corresponding state India pursues. Based on our above statement, the following stages and complementary policies could be considered in Fig 6. The changes of all output variables are shown in Table 11, and the overall changes in health are shown in Table 12.

**Table 11:** Transformation of the output value.

Stage	IN_3_	IN_4_	O_1_	O_2_	O_3_
India	0	.256	.567	.403	.501
Stage1	0	.256	.796	.305	.462
Stage2	0	.256	.891	.429	.983
Stage3	1	.256	.602	.914	.904
Stage4	1	1	.433	.881	.838

**Table 12:** Transition of the health value.

	India	China	Japan	Belgium	USA
Health	.351	.426	.423	.478	.716

*5*.*2*.*2*.*1*. *Catch up with China*. The primary gap between India and China lies in all input indicators, suggesting that the gap between these two states is exceptionally huge. Therefore, many issues are to be settled in this period, which means policies should consider various channels, including funding, infrastructures, equality, and sustainability. In this period, Indian institutions could consider expanding and increasing the social funding sources for HES, striving for government expenditure support, increasing the scale of non-formal education, and enhancing the reform process of discipline structure simultaneously to catch up towards China. After the transformation, all the input variables increase from the value of India to China. Consequently, the health of our systems improves from .351 to .426.

Conversely, the economic entity output decreases from .567 to .419, the social benefits increase from .403 to .530, and the admission and employment rate drop from .501 to .359. Although both the O_1_ and O_3_ decrease, the health of the entire system increases. Therefore, there is enough evidence to believe that social benefits are a vital factor in determining health.

*5*.*2*.*2*.*2*. *Catch up with Japan*. After the transition period of catching up with China, the gap between India and Japan lies only in infrastructure. Therefore, we propose the following policy further to accelerate the construction process of Indian higher education infrastructures. Institutions are supposed to invigorate campus assets and explore the advantages of R&D achievements. After this period, IN_2_, the infrastructure input, increases from .218 to .322, and the corresponding evaluation score decreases from .426 to .423. The O_1_, O_2_, O_3_ increase, yet the overall health evaluation score drops. Owing to the increase in infrastructure input, the whole country devotes more money to this area. The development of the infrastructure accelerates the spread and implementation of the development of technology. Therefore, all the above three output factors increase. However, the magnitude of the increase in social benefits output is relatively small comparing to the decrease in efficiency. Therefore, our health score decreases but only in a relatively small quantity.

*5*.*2*.*2*.*3*. *Catch up with Belgium*. The period of catching up with Japan would facilitate the India-HES level to a large extent. Simultaneously, there is still a long way to go for India to catch up with Belgium, mainly demonstrated in infrastructures, equality, and sustainability. For instance, Indian educational institutions could consider using the principle of leverage to use financial leasing, imposing gender equality in education projects, and accelerating the construction of high-quality teachers. After this transition, both IN_3_ and IN_4_ increase significantly, and the health scores increase from .423 to .478. Both O_1_ and O_3_ drop, yet O_2_ increases. Owing to the increased inequality input, people in the whole society become more aware of that inequality, which leads to a rise in the overall quality of individuals. Therefore, the O_2_ increases. Due to the increase in the sustainability input, according to economic intuition, there is a trade-off between economic growth and its value, which indicates that the higher its value, the lower its growth rate. Without changing the volume input, the result should bear the loss of the O_1_, which is the technological and economic output.

*5*.*2*.*2*.*4*. *Catch up with the United States*. The final stage for India is to exceed the United States when the gap is mainly represented in funding and infrastructure. In this period, funding through resource sharing and borrowing experience from the Build-Operate-Transfer (BOT) financing model would be the main concern for Indian institutions. After this transition, both IN_1_ and IN_2_ increase significantly, and the health scores increase from .478 to .716, and all the output variables increase. The increase in O_1_ is evident because the higher the tech/economy input, the higher its output. Without changing the education equality and sustainability factor, the result reaches a higher level of O_1_ (social benefits) and O_3_, which measures the educational return factor by increasing the educational volume. With the aggrandize quantity of money devoted to tech/econ and educational expenses, the number of people who would get tertiary education will increase considerably. Therefore, O_2_ will increase. With the significant increase in the money devoted to infrastructure input, more people will have the opportunity to get access to HES or get employed, leading to an increase in O_3_. Additionally, the health score increases significantly. Due to that, if the country already has a large volume, it is rational for them to pursue educational equality and sustainability. However, blindly following equality and sustainability may lead to the exact reverse results if the country does not have enough volume.

### 5.3. Path comparisons

Policy changes usually take time, and different indicators of policy changes have distinct difficulties. Therefore, this research assumes that after implementing the policy change, the national input indicators grow at a specific growth rate and remain unchanged after implementing the policy. According to economic theory, if an index develops at a fixed growth rate and the growth rate is assumed to be *m*, and the value of the index is expressed as *x*(*t*), like the following equation:

$$x\left(t\right)=x\left(0\right)\cdot e^{mt}$$ (19)

After entering the indicators through the initial state and the final state, the time required to complete the policy change is calculated according to the following process.


$$ln\frac{x\left(t\right)}{x\left(0\right)}=mt\Rightarrow t=\frac{\text{ln}\frac{x\left(t\right)}{x\left(0\right)}}{m}$$ (20)

Since our input variable significantly correlates with the country’s GDP, the country’s nominal GDP growth rate is chosen instead of *m* to solve problems to simplify our analysis. Therefore, the time for the full implementation of the policy change is shown in Table 13.

**Table 13:** Required time for the implementation of the policy.

Policy	India	Stage1	Stage2	Stage3	Stage4
Policy1	22.457	20.402	30.000	20.023	92.882
Policy2	52.179	2.701	15.561	18.095	91.535

Based on our estimation, the total time needed to transform from the current state to the proposed two strategies is 92.88 years and 91.54 years, which are almost the same between the two strategies. Realistically, this research decided on the second strategy as a policy change. The first path is unrealistic since there will be no country focusing solely on improving a factor without considering other factors. Concerning the second path, the entire process is divided into ten durations during the first transformation from India to China, each unit being five years. We expect to apply only one policy change for each term and eventually implement all those policy changes. This process tends to be much more realistic than the former since the likely policy is currently in practice in many states, such as China.

Although the years calculated for the complete transition might seem excessively long, this happens because this paper uses GDP growth rates as a proxy variable for the growth rates of all input variables. Meaningful growth rates for input variables are more likely to be much faster than GDP growth rates. Therefore, the actual years required to complete the process may be significantly shorter than the estimated value.

## 6. Conclusions

This research establishes a health assessment model toward India-HES, considering two dimensions of absolute health and relative health. The value of absolute health is obtained by reducing 19 indicators into two input and three output indicators through the TOPSIS method and a further PCA. Additionally, relative health is divided into three aspects: efficiency, equality, and sustainability measured by the DEA-Tobit Model, TOPSIS method, and Augmented Solow Model. The normalized weighting scores of absolute and relative health indicators are added, and eight states’ comprehensive ranking is obtained globally. The United States is far ahead of other countries. A typical developing country, India, has high efficiency and an absolute health score in education while still has much room for improvement inequality and sustainability. Applying our evaluation model, the Indian current score and the proposed score are .351 and .716.

Additionally, for the development of India-HES, two possible paths are proposed. The first is a single-target catching- up path, aiming to raise the four directly affecting variables in turn to the top-level globally. The second is the multiple-target catching-up path, intending to gradually emulate great states’ advantages and slowly realize the transformation of the India-China-Japan-Europe-the USA process. The two paths are compared, quantitatively evaluating the real-world impacts of different stages, and concluding that the multiple-target path is a better way to achieve the comprehensive development of India-HES. Four policy implementations are proposed in each period. In each period, the related policies are proposed meanwhile.

- Funding: Expanding and increasing the social funding sources for HES, driving funding through resource sharing.
- Infrastructures: Striving for government expenditure support, invigorate campus assets, and explore the advantages of R&D achievements, making use of the principle of leverage to make use of financial leasing and borrowing experience from the BOT financing model.
- Equality: Expanding the scale of non-formal education, imposing the project of gender equality in education.
- Sustainability: Enhancing the reform process of discipline structure, accelerating the construction of high-quality teachers.

Finally, this paper set up a transformation function considering the relationship between economic growth rates and our indicators by comparing the required time of the two paths, 92.8 years, and 91.5 years, respectively, thus demonstrating that changes are somewhat tricky. Three main contributions are as follows:

- This research is the first attempt to use research models such as TOPSIS, the DEA-Tobit Model, and the augmented Solow model for an overall assessment of the Indian educational system.
- This study establishes a health assessment model after evaluating the four perspectives: volume, efficiency, equality, and sustainability.
- This paper proposes a multiple-target catching up path to help achieve the comprehensive development of the higher education system in India (India-China-Japan-Europe-the USA).

However, there are two limitations of our work. First, in the health assessment model of HES, the setting of several parameters is relatively subjective. Therefore, the normalized comparison of different standards and dimensions would vary slightly when using distinct evaluation algorithms. Besides, considering the complexity of the government operating mechanism, the implementation of the policy may not be as idealized as in the model, resulting in a deviation in the evaluation of policy effectiveness and time cost of implementation.

## Supporting information

S1 Table The detailed data information of this research. (DOCX)
