# Supplementary material for: Sustainable development and health assessment model of higher education in India: A mathematical modeling approach
Source: PLoS One. 2021 Dec 28;16(12):e0261776. doi: 10.1371/journal.pone.0261776 (PMC8714109; doi:10.1371/journal.pone.0261776)
Supplement: S1 Table — (DOCX) [file pone.0261776.s001.DOCX]

| **S1 Table: The detailed data information in this research.** | | | | | |
| --- | --- | --- | --- | --- | --- |
| **Indicator** | **Sub-indicator** | **Description** | **Measurement** | **Database** | **Website** |
| Absolute health/Efficiency | Funding Investment IN_1_ | Public expenditure in tertiary education as a percentage of total expenditure | Direct download | World Bank | <https://data.worldbank.org.cn/indicator/SE.XPD.TOTL.GD.ZS?view=chart> |
|  | Funding Investment IN_1_ | Annual expenditure per student in all tertiary education | Direct download | OECD | <https://stats.oecd.org/Index.aspx?QueryId=58545> |
|  | Funding Investment IN_1_ | Gross Domestic expenditure on R&D | Direct download | OECD | <https://stats.oecd.org/Index.aspx?QueryId=15962> |
|  | Funding Investment IN_1_ | Teachers' starting salary | Direct download | OECD | <https://stats.oecd.org/Index.aspx?QueryId=108526> |
|  | Infrastructure/personnel investment IN_2_ | Students/Teachers | Direct download | World Bank | <https://data.worldbank.org.cn/indicator/SE.TER.ENRL.TC.ZS?view=chart> |
|  | Infrastructure/personnel investment IN_2_ | Researchers | Direct download | World Bank | <https://data.worldbank.org.cn/indicator/SP.POP.SCIE.RD.P6?view=chart> |
|  | Infrastructure/personnel investment IN_2_ | No. College | Find the number of colleges in QS 300 in the certain countries | QS 300 | <https://www.qschina.cn/> |
|  | Technology/economic entity output O_1_ | Triadic patent families | The growth rate of the expenditure on higher education | OECD | <https://stats.oecd.org/Index.aspx?QueryId=9118> |
|  | Technology/economic entity output O_1_ | GDP per capita | Direct download | OECD | <https://stats.oecd.org/Index.aspx?QueryId=66948> |
|  | Technology/economic entity output O_1_ | Real GDP growth rate | Direct download | OECD | <https://stats.oecd.org/Index.aspx?QueryId=350> |
|  | Social Benefits O_2_ | Work-life balance | Direct download | OECD | <https://stats.oecd.org/Index.aspx?QueryId=93029> |
|  | Social Benefits O_2_ | Bulling | Direct download | OECD | <https://stats.oecd.org/Index.aspx?QueryId=101794> |
|  | Social Benefits O_2_ | Social responsibility | Direct download | OECD | <https://stats.oecd.org/Index.aspx?QueryId=79337> |
|  | Admission and employment rate O_3_ | Employment rates | Direct download | OECD | https://stats.oecd.org/Index.aspx?QueryId=68400 |
|  | Admission and employment rate O_3_ | Enrollment rate (higher education) | Direct download | OECD | <https://data.worldbank.org.cn/indicator/SE.TER.ENRR?view=chart> |
|  | Admission and employment rate O_3_ | Foreign students in total tertiary education | Direct download | OECD | <https://stats.oecd.org/Index.aspx?QueryId=10265> |
|  | Admission and employment rate O_3_ | International students in total tertiary education | Direct download | OECD | <https://stats.oecd.org/Index.aspx?QueryId=108570> |
| Equality | Admission and employment rate | The enrollment rate difference for tertiary education by gender | Difference between different genders in enrollment rates | World Bank | <https://data.worldbank.org.cn/indicator/SE.ENR.TERT.FM.ZS?view=chart> |
|  | Social Benefits | Standard error of the number of 15-year-old student per school computer | Direct download and sort out the data we need | OECD | <https://stats.oecd.org/Index.aspx?QueryId=71925> |
|  | Admission and employment rate | Poverty Rate | Direct download | World Bank | <https://data.worldbank.org.cn/indicator/SI.POV.NAHC?view=chart> |
| Sustainability | Sustainability | Population growth rate | Derivative of population level | OECD | <https://stats.oecd.org/Index.aspx?QueryId=30383> |
|  | Sustainability | Real GDP growth rate | Direct download | OECD | <https://stats.oecd.org/Index.aspx?QueryId=350> |
|  | Sustainability | Human capital growth rate | The growth rate of the expenditure on higher education | World Bank | <https://data.worldbank.org.cn/indicator/SE.XPD.TOTL.GD.ZS?view=chart> |
| **Note:** GDP: Gross domestic product; R&D：Research and development; QS：Quacquarelli Symonds; OECD：Organization for Economic Co-operation and Development; Final accessed all the websites (November 15, 2021). | | | | | |
